# Models and Outcomes of Multidisciplinary Clinics in Colorectal Cancer

**DOI:** 10.3390/jcm13133815

**Published:** 2024-06-28

**Authors:** Seohyuk Lee, Kristen T. Crowell, Jessica A. Zerillo

**Affiliations:** 1 Department of Medicine, Beth Israel Deaconess Medical Center, Boston, MA 02215, USA; 2 Division of Colon and Rectal Surgery, Department of Surgery, Beth Israel Deaconess Medical Center, Boston, MA 02215, USA; 3 Harvard Medical School, Harvard University, Boston, MA 02115, USA; 4 Division of Medical Oncology, Department of Medicine, Beth Israel Deaconess Medical Center, Boston, MA 02215, USA

**Keywords:** colorectal cancer, multidisciplinary clinic, models, outcomes

## Abstract

Multidisciplinary clinics (MDCs) represent a potential platform through which high-quality, patient-centered care grounded in interdisciplinary expertise may be delivered for patients with colorectal cancer (CRC). This is increasingly important with the rapidly emerging diagnostic and treatment modalities as well as differential sequences of therapies available. MDCs have been reported to improve various outcomes across numerous non-colorectal cancers; however, data specific to the use of MDCs in CRC are more limited. In this report, we provide a narrative review of the different models of CRC MDCs in the literature and their associations with cancer care outcomes. We found significant heterogeneity in MDC operational logistics as well as reported outcomes across different practice settings. Further research is needed to better understand how MDCs may be optimally structured to meet the unique needs of patients with CRC and how they may affect CRC outcomes.

## 1. Introduction

Quality cancer care, as underscored by the American Society of Clinical Oncology (ASCO) and European Society for Medical Oncology (ESMO) consensus statement, is often rooted in the adoption of a multidisciplinary approach [1]. Various models of interdisciplinary cancer care have been developed and implemented across different practice settings [2,3]. In contrast to the more traditional tumor board in which the physician presenting a patient case typically formulates the treatment plan following consultative multidisciplinary discussion without the patient present, multidisciplinary clinics (MDCs) have emerged whereby specialists including those of medical, surgical, and radiation oncology contemporaneously meet the patient and coordinate a treatment plan in a single clinical encounter [4]. Importantly, given the growing incidence of colorectal cancer (CRC) among young adults [5] as well as emerging neoadjuvant treatment options [6,7], patient preferences may be heterogenous and patient participation is an integral aspect of MDCs in facilitating the development of a patient-centered treatment plan.

CRC remains the third most common cancer and third leading cause of cancer-related deaths in the United States (U.S.) [8]. With increasingly diverse diagnostic and treatment modalities as well as emerging differential sequences and even omissions of therapies for the management of locally advanced and metastatic CRC, interdisciplinary expertise is frequently necessitated in the development of a coordinated treatment plan [9,10,11,12]. However, a study of 14 National Cancer Institute Community Cancer Centers Program (NCCCP) sites found that less than half of patients with stage II or III colorectal cancer received care in settings identified as demonstrating high levels of case planning, the highest level of which was defined as patient care planning completely conducted via MDCs [2]. Furthermore, although MDCs have been reported to improve a variety of outcomes across numerous cancer types, including patient and physician satisfaction, time to initial treatment, and survival [13,14,15,16], data specific to MDCs in CRC are more limited.

Given that the available published data on CRC MDC models did not allow for a systematic review and meta-analysis, we provide here a narrative review of the different models of CRC MDCs and their associations with cancer care outcomes. Given the breadth of definitions applied to multidisciplinary care in the literature, we defined CRC MDCs as in-person or telehealth clinical meetings including the presence of a patient diagnosed with CRC and ≥2 physicians of different specialties involved in the patient’s oncologic care, reflecting the definition of optimal multidisciplinary care by the NCCCP as collaborative, prospective, and synchronous [17]. The focus of this review will be on those studies which have explicitly described the structures of their respective MDCs and met our aforementioned criteria.

## 2. Methods

PubMed was queried using a search strategy containing keywords related to CRC and MDCs. Supplementary Materials shows the exact search strategy employed. Search results were filtered to provide articles available in the English language and those conducted with human participants. In total, 553 citations were ultimately retrieved and reviewed for inclusion. A search was additionally performed of the abstract literature from the ASCO, ASCO Gastrointestinal Cancers, and ASCO Quality Care symposiums; however, no abstracts were ultimately included as none included explicit descriptions of their respective MDCs’ structures. Studies were examined for a variety of measures including structural (e.g., clinical volume, financial return), process (e.g., time to receipt of diagnostic studies, time to treatment), outcome (e.g., patient and referring or treating provider satisfaction, patient morbidity and mortality), and balance (e.g., care delays, overtreatment).

## 3. Results

### 3.1. CRC MDC Models

Seven studies related to MDCs in CRC met our inclusion criteria [18,19,20,21,22,23,24]. Among these, five included patients with colon or rectal cancer [18,21,22,23,24], one only those with rectal cancer [19], and one examined patients with various gastrointestinal cancers but with data for those with CRC separately delineated [20]. Except for a single study based out of South Korea [19], the remaining six studies examined patients in the U.S. Specialists from medical, surgical, and radiation oncology were involved in all seven MDCs. Four MDCs had available representatives from genetics, pathology, and radiology; three had specialists from gastroenterology and nutrition; and two included social work. With respect to clinic frequency, three MDCs were conducted weekly and one was scheduled as needed; the frequencies of the remaining three were not described. Table 1 summarizes the key characteristics of the seven MDCs described in the included studies.

#### 3.1.1. MDC Development

Three studies described the creation of their respective MDCs [18,21,23]. As reported by Vu et al. [18], the University of Michigan CRC MDC was formed in 2012 with input from representatives of each included clinical specialty, focusing on care coordination, patient-centeredness, quality improvement, research study accrual, and specialty engagement. The CRC MDC at William Beaumont Hospital was created in 2009 and benefited from having the necessary resources and specialties already well-established, thus requiring only logistical restructuring of care services [21]. In contrast to these in-person MDCs, a virtual MDC was developed based on a pre-existing in-person MDC at the Sinai Hospital of Baltimore in 2020 during the COVID-19 pandemic in response to social distancing restrictions [23].

#### 3.1.2. MDC Patient Referral

Six studies described the patient referral process for their respective MDCs [18,19,20,22,23,24]. The University of Michigan and University of Colorado Hospital MDCs had, respectively, all new CRC referrals directed to the clinic and all patients with a gastrointestinal malignancy evaluated for MDC candidacy [18,20]. Similarly, the University of Alabama at Birmingham MDC evaluated all patients with rectal cancer as well as complicated cases of colon cancer [22]. The remaining three clinics at Cleveland Clinic, Yonsei Cancer Center, and Sinai Hospital of Baltimore evaluated those patients referred to them by a physician [19,23,24].

#### 3.1.3. MDC Logistics

**Figure 1:**
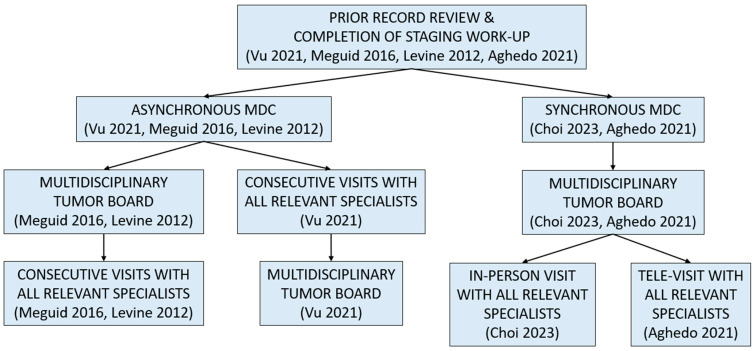
Summary of colorectal cancer multidisciplinary clinic logistics among included studies. Abbreviations: MDC = multidisciplinary clinic. References: Vu et al., 2021 [18], Choi et al., 2023 [19], Meguid et al., 2016 [20], Levine et al., 2012 [21], and Aghedo et al, 2021 [23].

Five studies described the specific logistics of their respective MDCs, summarized in Figure 1 [18,19,20,21,23]. Prior to the MDC sessions, clinics typically relied on a nurse coordinator to obtain and review patients’ prior diagnostic and treatment records as well as schedule appointments and additional necessary staging workup as indicated by national guidelines [18,20,21]. At the Sinai Hospital of Baltimore, this role was undertaken by the surgeon or a physician assistant [23].

MDCs commonly started with a multidisciplinary tumor board conference during which patients’ history and diagnostics, possible local or systemic treatment options, and trial eligibility were discussed by the relevant specialists [18,19,21,23]. During the MDC sessions themselves, two models of operation were observed across these studies: (1) asynchronous, whereby patients met with each relevant specialist separately in sequential order, and (2) synchronous, whereby patients met with all relevant specialists together.

At the William Beaumont Hospital, patients were scheduled to be seen following tumor board conferences via consecutive appointments with each relevant specialist through which coordinated treatment plans were ultimately developed [21]. As needed, patients could additionally consult with a clinical trial nurse, enterostomal therapist, genetics counselor, nutritionist, or social worker. Follow-up appointments, diagnostics, and treatments were scheduled by a nurse navigator. The University of Michigan and University of Colorado Hospital MDCs similarly operated in this asynchronous manner [18,20]. In the former, all patients younger than 50 years of age, with polyposis, or with a family history of CRC were scheduled for a genetics appointment. Specialties which were less frequently needed—including genetics, hepatobiliary and thoracic surgery, and radiation oncology—had appointments clustered for efficiency. A shared workroom for all providers facilitated ongoing discussions during the clinic.

In contrast, the MDCs at Yonsei Cancer Center and Sinai Hospital of Baltimore operated through the synchronous model [19,23]. In the tele-MDC described by Aghedo et al. [23], patients were scheduled to be seen in-person immediately following the tumor board conference by the surgeon, with the remaining specialists simultaneously engaged virtually via video teleconferencing. Following each specialist interviewing the patient in this group setting and discussing their respective roles in the patient’s care, the surgeon privately performed a physical examination and reported findings to the entire team. The final treatment plan was then formulated and discussed with the patient. The enhanced development of a therapeutic alliance via the presence of an in-person clinical team representative, ability to perform a physician exam, and minimization of potential patient barriers to technology access or use were highlighted strengths of this approach. The Yonsei Cancer Center MDC similarly had simultaneous involvement of multiple physicians with the goal of supporting patients’ ability to make informed decisions about interdisciplinary treatment plans [19].

### 3.2. CRC MDC Outcomes

Among the seven included studies, the reported outcomes primarily focused on structural and process factors. Only one study reported patient and provider satisfaction with the MDC experience [23], and no study evaluated the influence of MDCs on patient morbidity or mortality. No data regarding financial aspects or appropriate use of treatment (i.e., under- or over-utilization) of MDCs were reported in any study, though William Beaumont Hospital did report on whether diagnostic testing was consistent with guidelines [23].

#### 3.2.1. Service Utilization

Three studies reported utilization rates of CRC MDC services [18,19,21]. At the University of Michigan MDC, 1121 (65%), 1024 (60%), 220 (13%), and 205 (12%) patients, respectively, met with colorectal surgery, medical oncology, radiation oncology, and hepatobiliary surgery at the initial visit. A total of 967 (57%) patients saw only one specialist at the initial visit, 471 (28%) saw two specialists, 214 (13%) met with three specialists, and 57 (4%) saw four or more specialists. A clinical geneticist met with 369 (22%) patients at least once on any clinic visit. Patient volume increased more than 5-fold between clinic inception in 2012 and 2017 [18]. At the Yonsei Cancer Center CRC MDC, the mean number of clinic sessions conducted per patient was 1.6 (range, 1–6). The rate of patient referrals to the MDC increased from 11.7% in the first year of operations in 2012 to 27.1% in 2018 [19]. At the William Beaumont Hospital, patients receiving care at the MDC were significantly more likely to undergo consultation with medical oncology during the perioperative course relative to patients receiving care outside of the MDC (98.9% versus 61.5%, *p* < 0.0001). This finding persisted even when examining only those with non-stage I disease (98.6% versus 70.8%, *p* < 0.0001) [21].

#### 3.2.2. Patient and Physician Satisfaction

Measures of patient and physician satisfaction were reported only by Aghedo et al. [23] in reference to their tele-MDC model. Five-point Likert scale surveys modified from existing instruments validated in evaluating satisfaction with telemedicine services were conducted after each MDC session by both patients and physicians. Both groups reported high degrees of satisfaction with the care quality, communication, teamwork, and technological aspects of the MDC. Physicians expressed being highly open to incorporating a virtual MDC in their respective practices, and patients reported they would be interested in future virtual consultations and would recommend the virtual MDC to other patients. Physicians additionally expressed low concerns for medical errors arising from the virtual format.

#### 3.2.3. Diagnostic Outcomes

Four studies examined how MDCs influence diagnostic outcomes in patients with CRC (Table 2) [20,21,22,24]. Among patients with CRC being evaluated at the University of Colorado Hospital MDC, 16.3% experienced a change in clinical diagnosis, pathologic diagnosis, or staging upon interdisciplinary review. Additionally, 13.8% of patients experienced a change in cancer staging and 2.5% a change in pathology based on multidisciplinary review of radiographic or endoscopic results and pathology findings, respectively. No patients experienced a change in clinical diagnosis based on interdisciplinary review of radiographic evaluations; however, 6.2% of patients had an incidental finding unrelated to the primary diagnosis newly noted, including adrenal or breast nodules, benign cysts, colorectal thickening, prostatomegaly, and pulmonary emboli [20].

Relative to patients with colon cancer receiving care outside of an MDC, those receiving care at an MDC were found to more likely have fully completed diagnostic workup recommended by national guidelines prior to surgical resection (91.7% versus 27.5%, *p* < 0.0001). Similar findings were reported for patients with rectal cancer (84.0% versus 15.3%, *p* < 0.0001). With respect to individual preoperative evaluation parameters completed, patients with CRC at an MDC—relative to those not—were more likely to have completed CT imaging of the abdomen (97.5% versus 83.1%, *p* = 0.03) and chest (95.0% versus 37.1%, *p* < 0.0001) as well as undergone CEA (100% versus 63.8%, *p* < 0.0001) and microsatellite instability (29.6% versus 10.6%, *p* = 0.0001) testing. Compared to patients with rectal cancer not at an MDC, those at an MDC were additionally more likely to have completed a preoperative transrectal ultrasound (88.0% versus 37.7%, *p* < 0.0001) [21].

Conflicting findings were reported regarding time intervals for diagnosis-related processes among patients with CRC receiving care in MDC and non-MDC settings. At the University of Alabama, Birmingham, patients at the MDC versus those not experienced similar time intervals between diagnosis and completing staging workup (13 days versus 14 days, *p* = 0.48) and between diagnosis and initial clinic visit (14.5 days versus 18 days, *p* = 0.10) [22]. Patients at the Cleveland Clinic receiving care through the CRC MDC contrastingly experienced significantly shorter time intervals from diagnosis to initial contact (4.5 days versus 11 days, *p* < 0.001), diagnosis to appoinment scheduling (8.6 days versus 17 days, *p* = 0.01), and initial contact to initial visit (3 days versus 6 days, *p* = 0.02) relative to those receiving care outside of the MDC. However, the average time intervals from having no clinical staging workup to staging completion (11.0 days versus 11.4 days, *p* = 0.90) or having staging workup partially to fully completed (6.0 days versus 9.1 days, *p* = 0.30) were not significantly different between MDC and non-MDC patients [24].

#### 3.2.4. Treatment Outcomes

All seven studies evaluated how MDCs influence treatment outcomes among patients with CRC (Table 3) [18,19,20,21,22,23,24]. Among patients with CRC being evaluated at an MDC, 12.5% of patients experienced changes in treatment (i.e., surgical eligibility or approach, chemotherapy regimen, clinical trial eligibility) upon interdisciplinary review as compared to management previously suggested at an outside institution [20]. At the University of Michigan, 63% of patients received at least one treatment modality (surgery, chemotherapy, or radiation) through the CRC MDC, with 46% having undergone surgery and 30% having received chemotherapy through the MDC. In total, 5% of patients received all three treatment modalities [18]. At the Sinai Hospital of Baltimore, the average time between the virtual MDC session and initiation of definitive treatment was 30.9 days (standard deviation, 13.6 days) [23].

Relative to non-MDC CRC patients, MDC CRC patients were identified as undergoing more local and systemic therapies. At the Yonsei Cancer Center, CRC MDC patients underwent a greater number of radiotherapy sessions (1.3 versus 0.7, *p* = 0.004) and were more likely to undergo radiotherapy at all (63.6% versus 42.8%, *p* = 0.003). Similar findings were observed for the number of surgeries undergone (1.3 versus 0.9, *p* = 0.007). MDC patients were additionally more likely to experience a higher number of changes in systemic treatment regimens (2.5 versus 1.7, *p* < 0.001), receive capecitabine (57.6% versus 36.6%, *p* = 0.002), and receive a targeted agent (68.2% versus 54.2%, *p* = 0.044) [19]. At the William Beaumont Hospital, MDC CRC patients—relative to non-MDC CRC patients—were more likely to undergo neoadjuvant or adjuvant systemic treatment (62.5% versus 41.5%, *p* = 0.02). Similarly, MDC patients with rectal cancer were more likely to undergo neoadjuvant treatment compared to non-MDC patients (76% versus 20%, *p* < 0.0001), including when only considering non-stage I patients (82.6% versus 30.9%, *p* = 0.0001) [21].

Conflicting findings were reported regarding time intervals to treatment among patients with CRC receiving care in MDC and non-MDC settings. At the Cleveland Clinic, MDC CRC patients relative to non-MDC CRC patients experienced a significantly shorter time from initial visit to treatment initiation (19 days versus 27 days, *p* < 0.001), with only 22.9% of MDC patients having 30 or more days between the initial visit to treatment initiation compared to 42.9% of non-MDC patients (*p* = 0.05). However, the time from diagnosis to treatment initiation was not significantly different (40 days versus 40.5 days, *p* = 0.97) [24]. At the University of Alabama at Birmingham, MDC and non-MDC patients with CRC experienced similar time intervals from diagnosis to treatment initiation (30 days versus 37 days, *p* = 0.07), diagnosis to neoadjuvant treatment (30 days versus 34 days, *p* = 0.07), diagnosis to surgery (32.5 days versus 38.0 days, *p* = 0.35), initial visit to treatment initiation (14.5 days versus 16.0 days, *p* = 0.37), and completion of neoadjuvant treatment to surgery (63 days versus 64 days, *p* = 0.60). Subgroup analyses by race/ethnicity and insurance type did not show differences in time from diagnosis to treatment initiation. When examining time from diagnosis to treatment initiation by distance travelled by the patient from home to the care setting, non-MDC CRC patients experienced a significantly longer time interval with increased distance (*p* = 0.02), whereas MDC patients did not (*p* = 0.5) [22].

## 4. Discussion

With an evolving patient population and the advent of increasingly diverse treatment options and sequences for locally advanced and metastatic CRC, MDCs represent a potential platform through which high-quality, patient-centered cancer care grounded in interdisciplinary expertise may be delivered. However, our current understanding of different CRC MDC models is based on only a handful of studies additionally limited by significant heterogeneity in MDC operational logistics as well as reported outcomes which made performing a more robust systematic review and meta-analysis infeasible. CRC MDCs examined here varied widely in numerous aspects including the specialties involved, referral process, clinic logistics, and mode of delivery (in-person versus telehealth); however, they could be broadly categorized as asynchronous or synchronous based on whether patients met with relevant specialists, respectively, sequentially or altogether. Such care delivery models are similar to what has been reported for MDCs across different cancer types [3]. Given prior data suggesting MDC models may be more appropriate for specific cancer types [3], further research is needed to better understand how MDCs may be optimally structured to meet the unique needs of patients with CRC. There is additionally a need to identify potential patient, disease, and planned treatment characteristics that most benefit from the MDC mode versus other modalities of interdisciplinary care, such as tumor boards. It may be that the MDC model is most beneficial not only for specific patients but also for certain practice settings or institutions over others.

MDCs reflect a high-cost practice given the need for multiple providers to be simultaneously present with typically fewer patients overall being seen over the same time span. A prior study of various MDCs at a single comprehensive cancer center found that MDCs may lack adequate resources and infrastructure as compared with specialists operating in their respective specialty-specific clinics, resulting in fewer patients being seen [3]. To our knowledge, financial outcomes associated with CRC MDCs have yet to be examined but represent a highly necessary area of investigation to further our understanding of how CRC MDCs may be best modeled to achieve high-value care. Innovative approaches to the MDC model may help to address such resource constraints as well as make any value of multidisciplinary care more accessible to a broader reach of patients. As evidenced by the high degree of physician and patient satisfaction reported with the tele-CRC MDC at the Sinai Hospital of Baltimore [23], virtual MDCs may be one attractive option, especially in low-resource settings.

Understanding the impact of the typical in-person MDC—as well as any potential future innovative MDC models—on CRC outcomes remains a ripe area for research. The findings here are hypothesis-generating for the future design and prospective study of MDC models. Whether the improvements in guideline-adherent diagnostics or time to treatment with receipt of MDC care observed in some of the aforementioned studies translate to improvements in disease-specific or psychological outcomes for patients is yet unknown. Additionally, although it seems possible that the observed higher rates of guideline-concordant diagnostic testing associated with CRC MDC care may improve outcomes, this has yet to be specifically investigated. Receipt of more care modalities, however, should not be assumed to be a success of the MDC model and should be further examined to ensure that MDCs do not result in inappropriate overuse of diagnostic and therapeutic medical care. Moreover, studies have yet to directly evaluate differences in CRC treatment plans arising from a single-visit MDC versus separate specialty-specific clinics as well as patient comprehension of treatment options.

Importantly, there has yet to be an established definition for what comprises an MDC, and thus it is highly warranted for relevant accrediting agencies and professional organizations to develop consensus around this. Future investigations should clearly define the structures of their respective MDCs to facilitate comparisons across studies as well as encourage the development of new strategies for delivering interdisciplinary care.

## Figures and Tables

**Table 1:** Key characteristics of included colorectal cancer multidisciplinary clinics.

Author	Journal	Year	Cancer Type	Institution	Country	Cohort Size	Study Period	Specialties Involved	MDC Frequency	MDC Referral	MDC Logistics
Vu et al. [18]	American Journal of Surgery	2020	Colorectal	University of Michigan	USA	1711	5 years	MO, SO, RO, Pathology, Radiology, Gastroenterology, Genetics, Nutrition, Social Work	Weekly	All new colorectal cancer referrals evaluated by MDC	Dedicated scheduler retrieves previously completed or scheduled diagnostic/treatment records Dedicated nurse navigator reviews records, schedules required additional diagnostics and specialty provider visits using NCCN guideline-derived algorithms, and contacts patient prior to visit Patient evaluated by all specialty providers likely to be involved in management based on cancer staging in 1 day, providers work in a shared space to facilitate interdisciplinary communication Patient discussed at same-day tumor board with treatment plan developed and communicated to patient and referring physician
Choi et al. [19]	Cancer Medicine	2023	Rectal cancer	Yonsei Cancer Center	South Korea	66	Median follow-up from initial diagnosis: 68.0 months	MO, SO, RO	Scheduled as needed	Physician referral	Multidisciplinary discussion with involved providers with attempt to achieve consensus before patient inclusion Addition of patient to discussion
Meguid et al. [20]	Annals of Surgical Oncology	2016	Gastrointestinal cancers, including colorectal	University of Colorado Hospital	USA	160	11 months	MO, SO, RO, Pathology, Radiology, Gastroenterology	Unknown	All patients with a gastrointestinal cancer diagnosis evaluated for candidacy	Patient evaluated by Advanced Practice Provider for need for additional diagnostics Diagnostics, history, and physical, encounter with support services completed on the day of but prior to MDC Interdisciplinary conference between included specialty providers Patient visit with all specialists involved in care
Levine et al. [21]	International Journal of Colorectal Disease	2012	Colorectal	William Beaumont Hospital	USA	88	1 year	MO, SO, RO, Pathology, Radiology, Genetics, Nutrition, Social Work, Clinical Trial Coordinator, Enterostomal Therapy	Weekly	Unknown	Dedicated nurse navigator contacts patient, retrieves prior diagnostic/treatment information, and schedules additional diagnostics as indicated Interdisciplinary tumor board Consecutive visits between patient and relevant specialists with formulation of a treatment plan
Bajpai et al. [22]	American Journal of Surgery	2022	Colorectal	University of Alabama at Birmingham	USA	115	3 years	MO, SO, RO	Unknown	All rectal cancer and complex colon cancer patients	Unknown
Aghedo et al. [23]	Cureus	2021	Colorectal	Sinai Hospital of Baltimore	USA	18	Unknown	MO, SO, RO, Pathology, Radiology, Gastroenterology, Genetics, Nutrition, Primary Care	Weekly	Physician referral	Records review by surgeon and/or physician assistant with completion of any outstanding staging workup as indicated by NCCN guidelines prior to MDC Virtual interdisciplinary case review with development of preliminary diagnostic and treatment plan, only the surgeon is present in person at the MDC room Patient enters MDC room and meets rest of team virtually via video teleconferencing Each specialty provider interviews patient in group setting Surgeon conducts physical examination privately and presents findings to team Treatment plan formulated and discussed with patient
Kozak et al. [24]	Clinical Colorectal Cancer	2017	Colorectal	Cleveland Clinic	USA	35	22 months	MO, SO, RO, Genetics, Gynecology	Unknown	Physician referral	Unknown

Abbreviations: MDC = multidisciplinary clinic; USA = United States of America; MO = Medical Oncology; SO = Surgical Oncology; RO = Radiation Oncology; NCCN = National Comprehensive Cancer Network.

**Table 2:** Diagnostic outcomes associated with colorectal cancer multidisciplinary clinics.

Author	Measure	MDC	Non-MDC	*p*
Meguid et al. [20]	Diagnosis change following MDC review, No. (%)	26 (16.3)	-	-
	Staging change following MDC radiographic or endoscopic review, No. (%)	22 (13.8)	-	-
	Clinical diagnosis change following MDC radiographic review, No. (%)	0 (0)	-	-
	Pathology change following MDC review, No. (%)	4 (2.5)	-	-
	Incidental finding on MDC radiographic review, No. (%)	10 (6.2)	-	-
Levine et al. [21]	Receipt of perioperative medical oncology consultation, %	All patients: 98.9 Non-stage I patients only: 98.6	All patients: 61.5 Non-stage I patients only: 70.8	<0.0001 <0.0001
	Receipt of microsatellite instability testing, %	29.6	10.6	0.0001
	Preoperative CT abdomen completed, %	97.5	83.1	0.03
	Preoperative CT chest completed, %	95	37.1	<0.0001
	Preoperative CEA testing completed, %	100	63.8	<0.0001
	Preoperative transrectal ultrasound completed (rectal cancer only), %	88	37.7	<0.0001
	Diagnostic workup completed prior to resection, %	Colon: 91.7 Rectum: 84	Colon: 27.5 Rectum: 15.3	<0.0001 <0.0001
Bajpai et al. [22]	Time from diagnosis to staging workup completion, median, days	13	14	0.48
	Time from diagnosis to initial visit, median, days	14.5	18	0.1
Kozak et al. [24]	Time from diagnosis to initial contact, median (IQR), days	11 (7–23)	4.5 (1–10.75)	<0.001
	Time from initial contact to appointment scheduling, mean, days	4.9	6.2	0.35
	Time from diagnosis to appointment scheduling, mean, days	17	8.6	0.01
	Time from initial contact to initial visit, median (IQR), days	3 (2–4.5)	6 (3–8)	0.02
	Time from no clinical staging workup conducted to completion, mean, days	11	11.4	0.9
	Time from partial staging workup completed to full completion, mean, days	6	9.1	0.3
	Required ≥15 days to complete clinical staging workup, %	11.1	28.3	-

Abbreviations: MDC = multidisciplinary clinic; IQR = Interquartile Range.

**Table 3:** Treatment outcomes associated with colorectal cancer multidisciplinary clinics.

Author	Measure	MDC	Non-MDC	*p*
Vu et al. [18]	Receipt of ≥1 treatment modality * at MDC, No. (%)	1085 (63)	-	-
	Receipt of all 3 treatment modalities * at MDC, No. (%)	79 (5)	-	-
	Receipt of surgery at MDC, No. (%)	792 (46)	-	-
	Receipt of chemotherapy at MDC, No. (%)	510 (30)	-	-
Choi et al. [19]	Receipt of ≥1 radiotherapy sessions (%)	63.6	42.8	0.003
	Number of radiotherapy sessions per patient, mean (SD)	1.3 (1.5)	0.7 (1.2)	0.004
	Number of surgery sessions per patient, mean (SD)	1.3 (1.1)	0.9 (1.0)	0.007
	Number of surgery ± radiotherapy sessions per patient, mean (SD)	2.6 (2.0)	1.6 (1.6)	<0.001
	Number of changes in systemic treatment regimens, mean (SD)	2.5 (1.3)	1.7 (1.2)	<0.001
	Receipt of capecitabine, %	57.6	36.6	0.002
	Receipt of targeted agent, %	68.2	54.2	0.044
Meguid et al. [20]	Treatment recommendation changes following MDC review, No. (%)	20 (12.5)	-	-
Levine et al. [21]	Receipt of perioperative systemic treatment, %	All patients: 62.5 Non-stage I patients only: 77.1	All patients: 41.5 Non-stage I patients only: 59.1	0.02 0.002
	Receipt of neoadjuvant treatment (rectal cancer only), %	All patients: 76.0 Non-stage I patients only: 82.6	All patients: 20.0 Non-stage I patients only: 30.9	<0.0001 0.0001
Bajpai et al. [22]	Time from diagnosis to treatment initiation, median, days	30	37	0.07
	Time from diagnosis to neoadjuvant treatment initiation, median, days	30	34	0.07
	Time from diagnosis to surgery, median, days	32.5	38	0.35
	Time from neoadjuvant treatment completion to surgery, median, days	63	64	0.6
	Time from initial clinic visit to treatment initiation, median, days	14.5	16	0.37
	Time from diagnosis to treatment initiation among patients of Caucasian race only, median, days	32	35	0.17
	Time from diagnosis to treatment initiation among patients of racial/ethnic minority backgrounds only, median, days	28	41	0.19
	Time from diagnosis to treatment initiation among patients with any private/public insurance, median, days	30	37	0.12
	Time from diagnosis to treatment initiation among patients without insurance (self-pay/charity), median, days	23	38	0.17
	Predicted probability of time from diagnosis to treatment as a function of distance from home to hospital, *p*-value	0.5	0.02	-
Aghedo et al. [23]	Time from tele-MDC visit to definitive treatment initiation, mean (SD), days	30.9 (13.6)	-	-
Kozak et al. [24]	Time from initial visit to treatment initiation, median (IQR), days	19 (15–27)	27 (21–35)	<0.001
	Time from diagnosis to treatment initiation, median (IQR), days	40 (30.5–48)	40.5 (30–48.75)	0.97
	>30 days from initial visit to treatment initiation, %	22.9	42.9	0.05
	Receipt of first treatment within 3 weeks of initial visit, %	57.1	30	0.01

Abbreviations: MDC = multidisciplinary clinic, SD = Standard Deviation; IQR = Interquartile Range. * Treatment modalities included surgery, chemotherapy, and radiotherapy.

## Supplementary Materials

The following supporting information can be downloaded at: https://www.mdpi.com/article/10.3390/jcm13133815/s1, Search strategy.
