# Supplementary material for: Models and Outcomes of Multidisciplinary Clinics in Colorectal Cancer
Source: J Clin Med. 2024 Jun 28;13(13):3815. doi: 10.3390/jcm13133815 (PMC11242721; doi:10.3390/jcm13133815)
Supplement: Supplementary file 1 [file jcm-13-03815-s001.zip › jcm-3005209-supplementary.pdf]

("cancer care facilities"[MeSH Terms] OR "patient care team"[MeSH Terms] OR "mdc"[Text Word] OR "multidisciplinary care clinic*"[Text Word] OR "multidisciplinary cancer clinic"[Text Word] OR "cancer clinic"[Text Word] OR "multidisciplinary care team*"[Text Word] OR "multidisciplinary tumor board"[Text Word] OR "interdisciplinary communication"[MeSH Terms]) AND (“colorectal cancer”[Text Word] OR “colon cancer”[Text Word] OR “rectal cancer”[Text Word] OR “CRC”[Text Word])
